# Supplementary material for: Science in the cloud (SIC): A use case in MRI connectomics
Source: Gigascience. 2017 Mar 7;6(5):1–10. doi: 10.1093/gigascience/gix013 (PMC5467033; doi:10.1093/gigascience/gix013)

Manuscript Click here to download Manuscript main.pdf

Science In the Cloud (SIC): A use case in MRI Connectomics

A list of authors and their affiliations can be found at the end of the manuscript.

Abstract

Modern technologies are enabling scientists to collect extraordinary amounts of complex and sophisticated data across a huge range of scales like never before. With this onslaught of data, we can allow the focal point to shift towards answering the question of how we can analyze and understand the massive amounts of data in front of us. Unfortunately, lack of standardized sharing mechanisms and practices often make reproducing or extending scientific results very difficult. With the creation of data organization structures and tools which drastically improve code portability, we now have the opportunity to design such a framework for communicating extensible scientific discoveries. Our proposed solution leverages these existing technologies and standards, and provides an accessible and extensible model for reproducible research, called "science in the cloud" (SIC). Exploiting scientific containers, cloud computing and cloud data services, we show the capability to launch a computer in the cloud and run a web service which enables intimate interaction with the tools and data presented. We hope this model will inspire the community to produce reproducible and, importantly, extensible results which will enable us to collectively accelerate the rate at which scientific breakthroughs are discovered, replicated, and extended.

1 Introduction

Neuroscience is currently in a golden age of data and computation. Through recent technological advances [1], experimentalists can now amass large amounts of high quality data across essentially all experimental paradigms and spatiotemporal scales; such data are ripe to reveal the principles of brain function and structure. In fact, many public datasets and open-access data hosting repositories are going online [2].

Concurrent with this onslaught of data is a desire to run analyses, not just on data collected in a single lab, but also on other publicly available datasets. An assortment of tools have been developed around the community which solve a wide variety of computational challenges on all types of data, enabling difficult scientific questions to be answered. With the ability to perform analyses often dependent only upon access to data and code resources, neuroscience is now more accessible, with a lower barrier to entry.

However, there is no tool or framework that enables research to be performed and communicated in a way that lends itself to easy extensibility, much less reproducibility. Currently, re-performing and extending published analyses whether through new data or code is often unbearably difficult; (i) data may be closed-access; (ii) data may be organized in an ad hoc fashion; (iii) the code may be closed-source or undocumented; (iv) code may have been run with undocumented parameters and dependencies; (v) analyses may have run with specifically hardware compiled code. These properties make validating and extending scientific claims challenging.

We propose a solution in the form of a publicly documented and deployable cloud instance with a specific pipeline installed and configured to extend published findings; an implementation we simply term "science in the cloud," or, SIC (Latin for "thus was it written"). SIC instances have several fundamental components, as summarized in Figure 1. To address data access, we put data in the cloud. To address data organization, we utilize recently proposed data standards. To address closed source and undocumented, we generate interactive demonstrations. To address software and hardware dependencies, we utilize virtualization, automated deployment, and cloud computing. SIC puts these pieces together to create a computing instance launched in the cloud designed for not only producing reproducible research, but enabling easily accessible and extensible science for everyone.

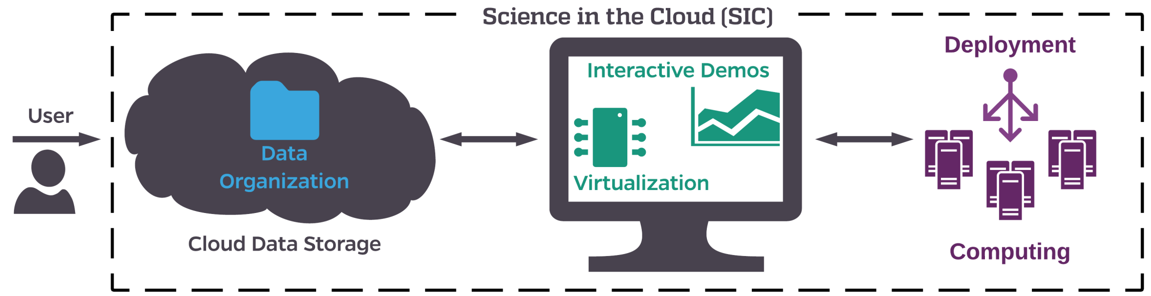

Figure 1: Framework for science in the cloud illustrating the six necessary components for SIC. Cloud data storage enables universal access to data products. Data organization structures enable consistent tool and users interactions across datasets. Interactive demonstrations allow users to participate in live scientific analyses. Virtualization enables tools to be deployed reliably and consistently. Deployment tools organize resources provided by computing platforms, and enable running analyses at scale. Together, these tools create a framework for discovery that is optimized for extensible science.

A focus on reproducibility is already commonplace in a variety of disciplines. In genomics, Bioboxes [3] provide a framework for reproducible and interchangeable analysis containers, and tools are exploiting scalable computing solutions and being published with reproduction instructions (see: [4; 5]). Commentaries on reproducible research provide suggestions to researchers on how to tackle the challenges that are present in their scientific setting [6; 7]. While these works have accelerated reproducibility and extensibility in their fields, the methods proposed do not scale to the cloud or enable real-time interactivity. SIC tackles these challenges for the burgeoning field of computational neuroscience, and provides a framework designed to minimize the bottlenecks between publication and novel discoveries.

We introduce and document an example use case of SIC with the ndmg pipeline, thus entitled SIC:ndmg. We have developed capability which enables users to launch a cloud instance and run a container which performs an analysis of a cohort of structural and diffusion magnetic resonance imaging scans by (i) downloading the required data from a public repository in the cloud, (ii) fully processing each subject's data to estimate a connectome for each

subject's associated graph statistics, and, optionally, (iii) plot quality control figures of various multivariate graph statistics.

2 Methods

There are six key decisions which must be made when following SIC: data storage, data organization, interactive demonstrations, virtualization, deployment, and computing. The selection made for each of these components will have a significant impact on available selections for the others. The final product will be a highly interdependent network of tools and data. Table 1 shows a summary of the selections made for each of the criteria enumerated in the previous section with rationales for the decisions. In general, the tools selected were those which provided the most command-line/Application Programming Interface (API) support for their service and had the most complete documentation or online support community, enabling setup with relative ease.

Cloud Data Storage There are several options when storing data in a publicly accessible location, such as a cloud storage service or public repositories. Depending on the nature of the data being stored, different concerns (such as privacy) must be satisfied. A service should also be accessible through an API, enabling developers to access the data programatically. Depending on the desired application, a systems autonomy is also a valuable feature, enabling the developer full control on how the data is organized. Amazon's S3 service was used in this SIC implementation because it satisfied all of these requirements. Similarly, Google's Cloud Engine or Microsoft Azure satisfy these requirements, and the decision was made based upon our existing domain knowledge and familiarity with each of these systems.

Data Organization The newly publicly-available data then needs to be organized in accordance with a data specification which enables users to navigate the repository successfully. Depending on the modality of data being used there are different structures which can be adopted. In the case of MRI, the BIDS [11] specification is a well documented and community developed standard which is intuitive and allows data to be both easily readable by humans and navigated by programs. Organizations such as "Neurodata without Borders" [12] would serve as an additional options for physiology data, but are unsuitable for this application. Formats such as MINC [13] focus heavily on metadata management but less on file hierarchy, making them useful though not fully sufficient for this application.

Interactive Demonstrations To encourage use of data and the tools used to analyze it, interactive demonstrations that enable users to visualize and work with some subset of the data are extremely valuable. Various programming languages have different types of demonstration environments available which either enable full interactivity or are pre-compiled to display code and results. A popular tool for interactive development and deployment of Python code is Jupyter [14], and thus was the tool used here. The popularity of this tool hopefully increases the average user's familiarity with the interface, lowering the barrier to entry

for interacting with SIC:ndmg. If the developer were more familiar with another programming language, there is no particular reason why one would select Jupyter over an equivalent package in R, such as R Notebook [15].

Virtualization To guarantee consistent dependencies and application setup, developing and distributing virtualized environments containing all necessary code products minimizes user effort to obtain expected performance. These virtual environments should be able to be deployed on any operating system and have minimal hardware dependent code. A key desiderata is that the virtualization system minimizes unnecessary overhead for the application. Though it does not affect run-time performance, a repository of public machine images is an attractive feature for this model as it enables sharing configurations. Docker [17] was chosen because it satisfies these practical requirements, and the accessibility of Docker Hub enables images to be quickly found and deployed. Virtual machines such as those created in Virtual Box [18] or VMware [19] provide lots of range in terms of operating systems which can be launched and allow native access to the machine through a GUI; while being great features,

Table 1: There are six key components which must be selected for SIC. **Bold** indicates the selections made here, with their positive and negative qualities compared to some alternatives.

Hurdles	Available Tools	Pros of Selection	Cons of Selection
1) Data Storage	**S3** [8], Dropbox [9], Google Drive [10]	API, pay-by-usage	requires user familiarity with Amazon tools
2) Data Organization	**BIDS** [11], NWB [12], MINC [13]	documented, validator, active community	new, not yet fully adopted
3) Interactive demo's	**Jupyter** [14], R Notebook [15], Shiny [16]	versatile, accessible	optimized for Python
4) Virtualization	**Docker** [17], Virtualbox [18], VMware [19]	lightweight, self-documented	–
5) Deployment	**manual**, ECS [20], Kubernetes [21], MyBinder [22], CBRAIN [23]	no additional dependencies	does not scale effectively
6) Computing	**EC2** [20], Google Compute Engine [24], Microsoft Azure [25]	scalable, flexible	requires technological expertise

it is unnecessary for this application.

Deployment Deployment platforms allow users to define a specific set of instructions that can be launched on a single or multiple machines simultaneously. In physical hardware configurations, a cluster's scheduler would play this role; in the cloud, such tools should be able to take advantage of compute resources across different locations and services, and enable scaling with the amount of processing required. In the case of SIC:ndmg, a single deployment was needed, so this challenge simply solved with manual deployment of the service. If multiple deployments were needed, either Kubernetes [21] or Amazon's ECS serve as scalable solutions. Tools such as CBRAIN [23] and MyBinder [22] also enable distributed deployment of code, but are more specialized in the requirements the tools and services that can be launched.

Computing Cloud computing services enable users to launch customized machines with specific hardware configurations and specifications, making them versatile for different varieties and scales of analyses. The more general the hardware that can be used, the more accessible the tool is for a user to adapt and use in their own environment. With no specific hardware requirements in this application, and previous in-house experience with the service, Amazon's EC2 was selected. The benefit of using EC2 is that deploying code at different scales and locations is trivially extendable, so implementations can be easily taken from prototype to deployment.

Further details of our specific implementation and methods are provided in Appendix A.

3 Results

We demonstrate a working example of SIC, SIC:ndmg. The ndmg pipeline [26] is an open-source, scalable pipeline for human structural connectome estimation from diffusion and structural MR images (collectively refer to hereafter as "multimodal MRI", or M3RI for brevity). The result is a portable and easily extensible tool for scalable connectome generation. A live demonstration is presented that enables reader interaction with the pipeline at the cost of a simple url click, and data products of the tool are presented in both the context of 'reproducibility' and 'extensibility.' This tool enables quantitative structural analyses of the human brain to be performed on populations of M3RI scans, and can lead to discoveries of the relationship between brain connectivity and neurological disease.

3.1 Neuroscience as a Service

The analysis transforms "raw" M3RI data into graphs. Kiar et al., (in preparation) describes the pipeline in detail; here we provide a brief overview. The pipeline (Figure 2) consists of four main steps: registration, tensor estimation, tractography, and graph estimation. Note that the choices below are made for expediency and simplicity, other choices might be beneficial depending on context.

Registration in ndmg is performed in several stages, and is all performed using FSL [27]. First, the diffusion image is self-aligned and noise corrected using the `eddy_correct` func-

tion. Second, the transform is computed which aligns the B0 volume of the diffusion image to the structural scan using `epi_reg`. Third, the transform between the structural image and a reference atlas is computed with `flirt`. Finally, the transforms are combined and applied to the self-aligned diffusion image. The tensor estimation and tractography steps are performed with the DiPy package [28]. A simple tensor model fits a 6-component tensor to the image, and deterministic tractography with the EuDx algorithm is run, producing a set of streamlines. Graph generation takes as input the fiber streamlines, and maps them to regions of interest (ROIs) defined by a pre-built parcellation (such as those packaged with FSL or generated with brain segmentation algorithms) and returns a ROI-wise connectome. An edge is added to the graph for each pair of nodes along a given fiber. The final step is computing (multivariate) graph statistics on the estimated connectomes. The statistics computed are [29]: number of non-zero edges, degree distribution, eigen sequence, locality-statistic 1, edge weight distribution, clustering coefficient, and betweenness centrality. These statistics provide insight into the structure of the brain graphs, and provide a low-dimensional feature by which the graphs for different scans can be compared to one another. To provide a preliminary quality control step, we plot the graph statistics [29] for each graph (Figure 4).

3.2 Live Demonstration

A demonstration of SIC:ndmg is available at `http://scienceinthe.cloud/`. This SIC is hosted on an EC2 micro-instance – it is very affordable, so can stay online indefinitely with little cost or maintenance. This instance is running a Jupyter server which contains the demonstration notebook, `sic_ndmg.ipynb`. Launching this demonstration notebook will pull up an interface which resembles that of Figure 3A.

For demonstration purposes, a downsampled subject is used in this notebook which reduces analysis time from ∼1 hr/subject/core to ∼3 min/subject/core. The ndmg pipeline has two levels of analysis: graph generation and computing summary statistic. Graph genera-

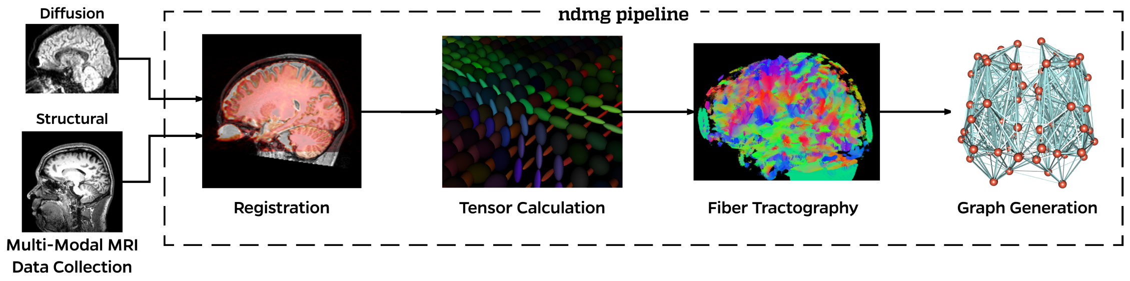

Figure 2: Structure of the ndmg pipeline connectome estimation. Taking as input diffusion and T1 weighted MRI, ndmg first aligns the diffusion data to a reference atlas by means of the T1 image. Tensors are then computed from the aligned diffusion volume. Fiber streamlines are generated by performing tractography on the tensors. Finally, the fibers are mapped between regions of interest (ROIs) which then become nodes in the graph.

tion is the process of turning diffusion and structural MR images into a connectome (i.e. brain graph), and the summary statistic computation produces a graph of several graph features on each produced connectome and plots them together. Running through the notebook (Figure 3A) chronologically will produce the brain graph, display the graph (Figure 3B), compute summary statistics (Figure 3C), and then plot the statistics.

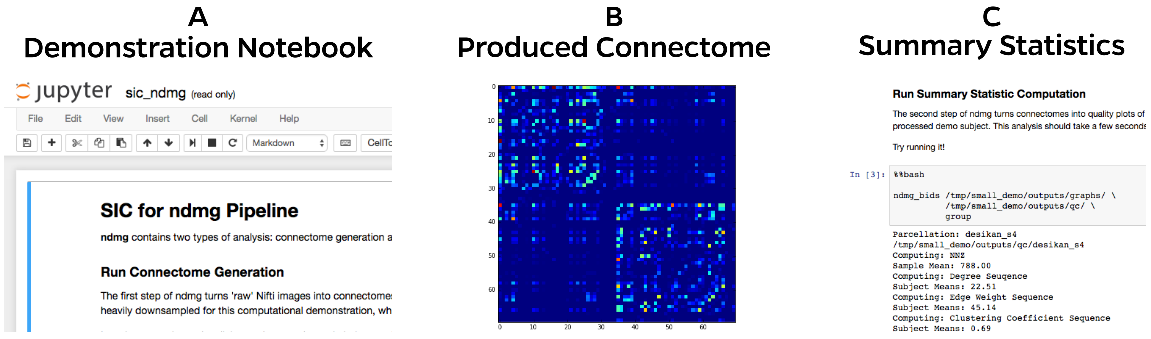

Figure 3: States of the demonstration notebook in the cloud. **A**) A Jupyter notebook displaying descriptions and code snippets to be run for both connectome estimation and summary statistic computation. **B**) After running connectome generation an adjacency matrix will appear to provide a visualization. **C**) Summary statistic computation calculates several graph features and plots them in a multipanel figure. The demonstration notebook is running version `v0.0.38` of ndmg.

3.3 Reproducible Results

In addition to the live demonstration, SIC:ndmg was used on the NKI1 [30] dataset consisting of 40 scans. Instructions on setting up a computer—in the cloud or locally—and running this analysis can be found in Appendix A. The NKI1 dataset is made publicly available through CORR [30], but has been organized in accordance to the BIDS [11] specification and re-hosted on our public S3 bucket, `mrneurodata`. The dataset consists of MPRAGE, DWI, and fMRI scans, where each subject has been scanned at least twice for each modality. More information about the subjects in this dataset and the scanning parameters used can be found on the CORR website[1].

Running the Docker-hosted scientific container `bids/ndmg:v0.0.37-1` on the NKI1 dataset for both on an EC2 m4.xlarge instance running Ubuntu 14.04 produced Figure 4, costing under $10. Table 2 summarizes the parameters used as inputs to SIC:ndmg to generate the graphs. Figure 4 provides insight into the variance of the dataset by a variety of different metrics. According to published work on these summary statistics [29], this dataset and pipeline combination produces expected results. A key benefit of this visualization is that it has high information density, showing us distributions for a variety of features for a large number of graphs, as opposed to more-common 1-dimensional features [31]. This figure was produced by the parameters summarized in Table 3.

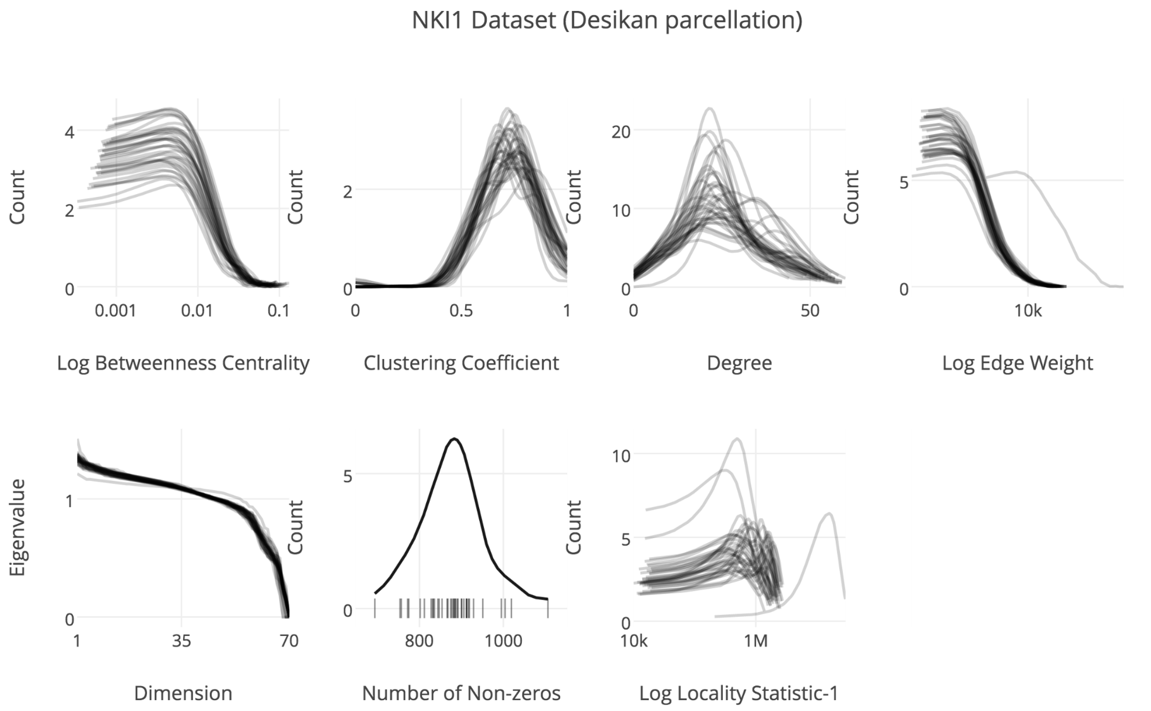

Figure 4: Running SIC:ndmg on the NKI1 dataset produces plots of graph statistics. Shown clockwise from top left are betweenness centrality distribution, clustering coefficient distribution, degree distribution, edge weight distribution, eigen sequence, number of non-zero edges, and the locality statistic-1 distribution of the graphs. The displayed summary statistics were computed on the graphs generated with the Desikan parcellation, using the `bids/ndmg:v0.0.37` Docker image.

Table 2: Command line arguments for connectome generation

Parameter	Value
data input directory	`/data/raw`
data output directory	`/data/connectome`
analysis level	`participant`
bucket name	`mrneurodata`
path on bucket	`NKI24`

The demonstration in the previous section executed the exact same pipeline and version as was run to generate Figure 4. The sole difference between execution of the demonstration and this implementation —aside from the data being processed—is that the demonstration ran the pipeline natively on an Ubuntu 14.04 machine rather than in the Docker image. The reason for this difference is that the demonstration uses a spatially downsampled set of atlases, different to those contained within the Docker image. These atlases are not input parameters for the

container, but are abstracted from the user for simplicity.

Table 3: Command line arguments for summary statistic computation.

Parameter	Value
data input directory	/data/connectome/graphs
data output directory	/data/qc
analysis level	group

3.4 Extensible Results

A crucial property of SIC is the simplicity it affords users to perform extensible science. Extensibility in this context can occur on several levels, including changing or adding (i) data, (ii) analyses, or (ii) visualizations. Figure 5 shows an example of such extensibility. A different dataset, the KKI2009 dataset [32], was processed using modified code, plotting the degree distribution on a log scale, with an additional plot added for cumulative variance analysis. The container used for this analysis on Docker hub is `bids/ndmg:v0.0.37-1`. Further details and instructions about how to extend SIC:ndmg specifically are available in Appendix B.

4 Discussion

The the SIC framework does not need to be confined to standalone instances and containers. With further work, this concept can be integrated into a platform in which users are able to launch a variety of analyses on a variety of datasets. A web browser interface which launches cloud containers performing the computation would make this a particularly accessible model, and would drastically improve the feedback loop between a scientist and their peers. This enables analyses to be easily replicated and refined, thus expediting scientific discovery. Tools such as Binder [22] accomplish this beautifully for Python, but the benefits of SIC are that this model can be applied not only to any containerizable applications, but big-data as well.

The selections made in SIC:ndmg regarding the six technological components highlighted above were chosen based on what we perceived to be most widely used and supported in the active online community. Other tools enumerated in Table 1 provide alternative features which can make SIC instances developed separately appear and run quite differently, but ultimately provide a comparable experience for the user. For instance, the decision to store data independently from a public repository (such as NITRC [33], LONI's IDA [34], LORIS [35], or ndstore [36]) leaves the onus of data organization on the developer rather than the repository, but in either case the user is able to access the data they need. This decision was made so that the developer would have complete control over their data and implementation. However, hosting data within these environments would have the advantage of enabling use of the infrastructure already built to support these platforms, such as performing meta-analyses and tracking provenance of the data itself, and is an exciting avenue for future work.

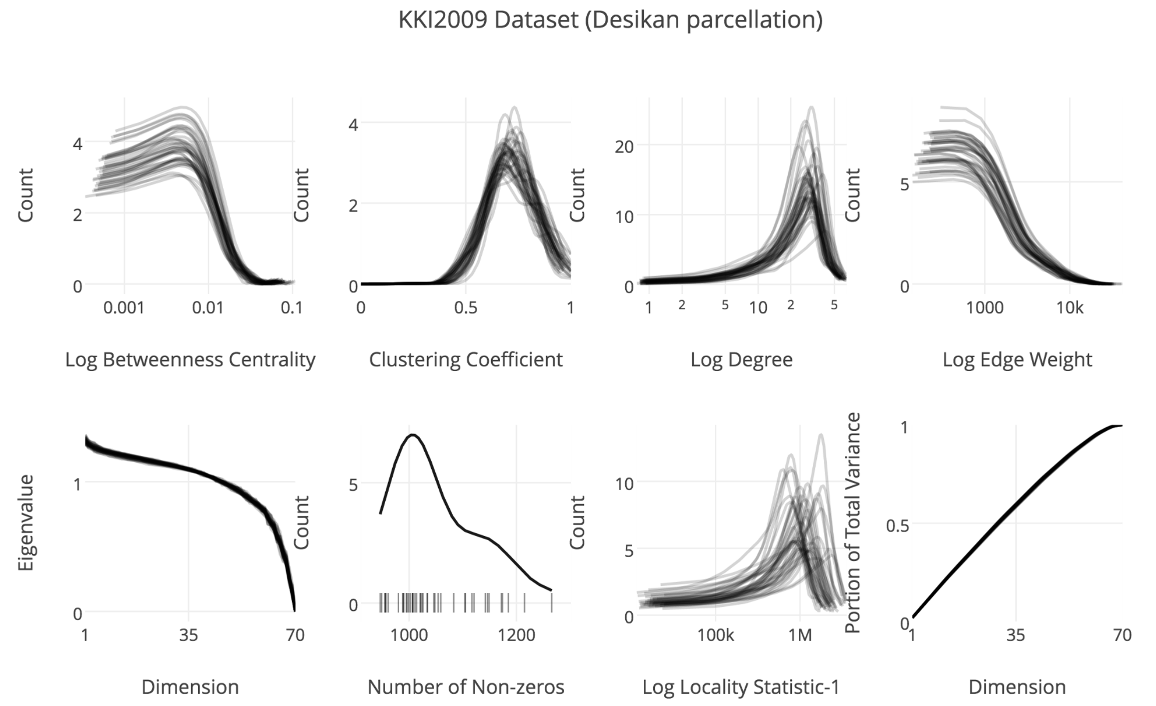

Figure 5: Analyses performed with code developed under the SIC framework are easily extensible. The extensions made between this plot and Figure 4 are a) using a different dataset, in this case KKI2009; b) adjusting existing plotting code in order to improve visualization of vertex degree in a log scale; and, c) adding cumulative variance analysis of the graph. The displayed summary statistics were computed on the graphs generated with the Desikan parcellation, using the `bids/ndmg:v0.0.37-1` Docker image.

Alternative deployment tools, such as Kubernetes, are attractive options as they provide clear visualizations of running processes, process versions, and would help enable SIC to scale well when working with big-data or running many parallel jobs.

This project stemmed from a sequence of three different initiatives. First, the Global Brain Workshop[2] brought together a collection of $60+$ scientists who converged on a set of grand challenges for global brain sciences. There was universal agreement that a global framework [37] would be instrumental in transitioning neuroscience from a data deluge to a data delight. Then, at the Open Data Ecosystem for Neurosciences[3], the working group on reproducibility decided that an example of a reproducible and extensible framework would be highly informative for ourselves and the greater community. Finally, the inaugural Stanford Center for Reproducible Neuroscience Coding Sprint[4] brought leaders in neuroimaging from around the globe to chart a path forward with standardizing a process for containerizing both open- and closed-source tools [38].

In summary, the SIC framework presents a standard of reliability and extensibility for scientific data distribution and analysis. SIC is an important building block towards a global scientific community, regardless of scientific discipline, and provides a practical implementation

of the idiom that science is done by “standing on the shoulders of giants.”

Author Information

Gregory Kiar[1,2], Krzysztof J. Gorgolewski[3], Dean Kleissas[4], William Gray Roncal[4,5], Brian Litt[6,7], Brian Wandell[3,8], Russel A. Poldrack[3], Martin Wiener[9], R. Jacob Vogelstein[10], Randal Burns[5], Joshua T. Vogelstein[1,2]

Corresponding Author: Joshua T. Vogelstein <jovo@jhu.edu>

[1]Department of Biomedical Engineering, Johns Hopkins University, Baltimore, MD, USA.
[2]Center for Imaging Science, Johns Hopkins University, Baltimore, MD, USA.
[3]Department of Psychology, Stanford University, Stanford, CA, USA.
[4]Johns Hopkins University Applied Physics Lab, Columbia, MD, USA.
[5]Department of Computer Science, Johns Hopkins University, Baltimore, MD, USA.
[6]Department of Bioengineering, University of Pennsylvania, Philadelphia, PA, USA.
[7]Department of Neurology, Hospital of the University of Pennsylvania, Philadelphia, PA, USA.
[8]Center for Cognitive and Neurobiological Imaging, Stanford University, Stanford, CA, USA.
[9]Department of Psychology, George Mason University, Fairfax, VA, USA.
[10]Intelligence Advanced Research Projects Activity (IARPA), McLean, VA, USA.

Declarations

Competing Interests The authors declare no competing interests in this manuscript.

References

[1] S. Grillner et al., “Worldwide initiatives to advance brain research,” Nature neuroscience, vol. 19, no. 9, pp. 1118–1122, 2016.

[2] R. A. Poldrack and K. J. Gorgolewski, “Making big data open: data sharing in neuroimaging,” Nature neuroscience, vol. 17, no. 11, pp. 1510–1517, 2014.

[3] P. Belmann, J. Dröge, A. Bremges, A. C. McHardy, A. Sczyrba, and M. D. Barton, “Bioboxes: standardised containers for interchangeable bioinformatics software,” GigaScience, vol. 4, no. 1, p. 1, 2015.

[4] A. Bremges, I. Maus, P. Belmann, F. Eikmeyer, A. Winkler, A. Albersmeier, A. Pühler, A. Schlüter, and A. Sczyrba, “Deeply sequenced metagenome and metatranscriptome of a biogas-producing microbial community from an agricultural production-scale biogas plant,” GigaScience, vol. 4, no. 1, p. 1, 2015.

[5] M. E. Aranguren and M. D. Wilkinson, “Enhanced reproducibility of sadi web service workflows with galaxy and docker,” GigaScience, vol. 4, no. 1, p. 1, 2015.

[6] S. R. Piccolo, A. B. Lee, and M. B. Frampton, “Tools and techniques for computational reproducibility,” bioRxiv, p. 022707, 2015.

[7] R. D. Peng, “Reproducible research in computational science,” Science, vol. 334, no. 6060, pp. 1226–1227, 2011.

[8] “Amazon simple storage service,” https://aws.amazon.com/documentation/s3/, accessed: 2016-10-10.

[9] “Dropbox,” https://dropbox.com, accessed: 2016-10-10.

[10] “Google drive,” https://drive.google.com/, accessed: 2016-10-10.

[11] K. Gorgolewski, T. Auer, V. Calhoun, C. Craddock, S. Das, E. Duff, G. Flandin, S. Ghosh, T. Glatard, Y. Halchenko et al., “The brain imaging data structure, a format for organizing and describing outputs of neuroimaging experiments.”

[12] J. L. Teeters, K. Godfrey, R. Young, C. Dang, C. Friedsam, B. Wark, H. Asari, S. Peron, N. Li, A. Peyrache et al., "Neurodata without borders: creating a common data format for neurophysiology," Neuron, vol. 88, no. 4, pp. 629–634, 2015.

[13] R. D. Vincent, A. Janke, J. G. Sled, L. Baghdadi, P. Neelin, and A. C. Evans, "Minc 2.0: a modality independent format for multidimensional medical images," in 10th Annual Meeting of the Organization for Human Brain Mapping, vol. 2003, 2004, p. 2003.

[14] "Project jupyter," http://jupyter.org/, accessed: 2016-09-10.

[15] RStudio, "R notebooks," 2016. [Online]. Available: http://rmarkdown.rstudio.com/r_notebooks.html

[16] R.-S. Server, "Rstudio: official website," 2015. [Online]. Available: http://shiny.rstudio.com/

[17] D. Merkel, "Docker: lightweight linux containers for consistent development and deployment," Linux Journal, vol. 2014, no. 239, p. 2, 2014.

[18] J. Watson, "Virtualbox: bits and bytes masquerading as machines," Linux Journal, vol. 2008, no. 166, p. 1, 2008.

[19] M. Rosenblum, "Vmware's virtual platform™," in Proceedings of hot chips, vol. 1999, 1999, pp. 185–196.

[20] "Amazon elastic compute cloud," http://docs.aws.amazon.com/AWSEC2/latest/APIReference/Welcome.html, accessed: 2016-11-01.

[21] E. A. Brewer, "Kubernetes and the path to cloud native," in Proceedings of the Sixth ACM Symposium on Cloud Computing. ACM, 2015, pp. 167–167.

[22] "Binder," http://mybinder.org/, accessed: 2016-09-10.

[23] T. Sherif, P. Rioux, M.-E. Rousseau, N. Kassis, N. Beck, R. Adalat, S. Das, T. Glatard, and A. C. Evans, "Cbrain: a web-based, distributed computing platform for collaborative neuroimaging research," Recent Advances and the Future Generation of Neuroinformatics Infrastructure, p. 102, 2015.

[24] S. Krishnan and J. L. U. Gonzalez, "Google compute engine," in Building Your Next Big Thing with Google Cloud Platform. Springer, 2015, pp. 53–81.

[25] "Microsoft azure: Cloud computing platform and services," https://azure.microsoft.com/en-us/, accessed: 2016-10-30.

[26] G. Kiar, W. Gray Roncal, D. Mhembere, E. Bridgeford, R. Burns, and J. Vogelstein, "ndmg: Neurodata's mri graphs pipeline," Aug. 2016. [Online]. Available: http://dx.doi.org/10.5281/zenodo.60206

[27] M. Jenkinson, P. Bannister, M. Brady, and S. Smith, "Improved optimization for the robust and accurate linear registration and motion correction of brain images," Neuroimage, vol. 17, no. 2, pp. 825–841, 2002.

[28] E. Garyfallidis, M. Brett, B. Amirbekian, A. Rokem, S. Van Der Walt, M. Descoteaux, and I. Nimmo-Smith, "Dipy, a library for the analysis of diffusion mri data," Frontiers in neuroinformatics, vol. 8, p. 8, 2014.

[29] D. Mhembere, W. G. Roncal, D. Sussman, C. E. Priebe, R. Jung, S. Ryman, R. J. Vogelstein, J. T. Vogelstein, and R. Burns, "Computing scalable multivariate glocal invariants of large (brain-) graphs," in Global Conference on Signal and Information Processing (GlobalSIP), 2013 IEEE. IEEE, 2013, pp. 297–300.

[30] X.-N. Zuo, J. S. Anderson, P. Bellec, R. M. Birn, B. B. Biswal, J. Blautzik, J. C. Breitner, R. L. Buckner, V. D. Calhoun, F. X. Castellanos et al., "An open science resource for establishing reliability and reproducibility in functional connectomics," Scientific data, vol. 1, p. 140049, 2014.

[31] R. C. Craddock, S. Jbabdi, C.-G. Yan, J. T. Vogelstein, F. X. Castellanos, A. Di Martino, C. Kelly, K. Heberlein, S. Colcombe, and M. P. Milham, "Imaging human connectomes at the macroscale," Nature methods, vol. 10, no. 6, pp. 524–539, 2013.

[32] B. A. Landman, A. J. Huang, A. Gifford, D. S. Vikram, I. A. L. Lim, J. A. Farrell, J. A. Bogovic, J. Hua, M. Chen, S. Jarso et al., "Multi-parametric neuroimaging reproducibility: a 3-t resource study," Neuroimage, vol. 54, no. 4, pp. 2854–2866, 2011.

[33] X.-z. J. Luo, D. N. Kennedy, and Z. Cohen, "Neuroimaging informatics tools and resources clearinghouse (nitrc) resource announcement," Neuroinformatics, vol. 7, no. 1, pp. 55–56, 2009. [Online]. Available: http://dx.doi.org/10.1007/s12021-008-9036-8

[34] J. D. Van Horn and A. W. Toga, "Is it time to re-prioritize neuroimaging databases and digital repositories?" Neuroimage, vol. 47, no. 4, pp. 1720–1734, 2009.

[35] S. Das, A. P. Zijdenbos, D. Vins, J. Harlap, and A. C. Evans, "Loris: a web-based data management system for multi-center studies," Frontiers in neuroinformatics, vol. 5, p. 37, 2012.

[36] R. Burns, K. Lillaney, D. R. Berger, L. Grosenick, K. Deisseroth, R. C. Reid, W. G. Roncal, P. Manavalan, D. D. Bock, N. Kasthuri et al., "The open connectome project data cluster: scalable analysis and vision for high-throughput neuroscience," in Proceedings of the 25th International Conference on Scientific and Statistical Database Management. ACM, 2013, p. 27.

[37] J. T. Vogelstein, K. Amunts, A. Andreou, D. Angelaki, G. Ascoli, C. Bargmann, R. Burns, C. Cali, F. Chance, M. Chun, G. Church, H. Cline, T. Coleman, S. de La Rochefoucauld, W. Denk, A. Belén Elgoyhen, R. E. Cummings, A. Evans, K. Harris, M. Hausser, S. Hill, S. Inverso, C. Jackson, V. Jain, R. Kass, B. Kasthuri, K. Kording, S. Koushika, J. Krakauer, S. Landis, J. Layton, Q. Luo, A. Marblestone, D. Markowitz, J. McArthur, B. Mensh, M. Milham, P. Mitra, P. Neskovic, M. Nicolelis, R. O'Brien, A. Oliva, G. Orban, H. Peng, A. Picchini-Schaffer, M. Picciotto, J.-B. Poline, M.-m. Poo, A. Pouget, S. Raghavachari, J. Roskams, T. Sejnowski, F. Sommer, N. Spruston, L. Swanson, A. Toga, R. J. Vogelstein, R. Yuste, A. Zador, R. Huganir, and M. Miller, "Grand Challenges for Global Brain Sciences," ArXiv e-prints, Aug. 2016.

[38] K. J. Gorgolewski, F. Alfaro-Almagro, T. Auer, P. Bellec, M. Capota, M. Chakravarty, N. W. Churchill, R. C. Craddock, G. Devenyi, A. Eklund, O. Esteban, G. Flandin, S. Ghosh, J. S. Guntupalli, M. Jenkinson, A. Keshavan, G. Kiar, P. R. Raamana, D. Raffelt, C. J. Steele, P.-O. Quirion, R. E. Smith, S. Strother, G. Varoquaux, T. Yarkoni, Y. Wang, and R. Poldrack, "Bids apps: Improving ease of use, accessibility and reproducibility of neuroimaging data analysis methods," bioRxiv, 2016. [Online]. Available: http://biorxiv.org/content/early/2016/10/05/079145

Appendix A Reproduction Instructions

Outlined here are the required steps to reproduce the results demonstrated. In the command blocks which follow, all commands preceded by a $ should be executed on the system which will run the code, with the exception of Section A.1.1, which provide instructions for how to start and connect to a cloud instance. Commands which are executed in a single line but were too long to fit on the page end with \ and are carried over to lines which have been indented. Lines beginning without any of these strings or indentations indicate outputs of previous commands. Below, the assumption is that the commands are being executed on a Unix based machine with access to a terminal. If being executed on Windows, installing a GNU environment such as Cygwin[5] will enable the user to have a similar experience.

A.1 Setting up Your Machine

Depending on whether or not one has local compute resources available, the cloud is a very appealing option for running resource-demanding computations spuriously. Depending on where running this service, in the cloud or locally, a slightly different set of instructions apply due to uncertainty about the user's operating system and Amazon Machine Images (AMIs) which come with preloaded dependencies for cloud instances.

A.1.1 Amazon EC2

The general procedure for launching any EC2 instance is as follows: select a base image, choose instance parameters (i.e. type, storage, security, access keys), launch, and connect[6]. Table 4 enumerates the specific choices that should be followed – for all others, default values are sufficient.

Table 4: Key selections for starting Amazon EC2 instance.

Instance Parameter	Value
Image	Amazon ECS-Optimized Amazon Linux AMI from AWS Marketplace
Type	m4.large
Storage	Root SSD volume to 100 GB

Once the instance has been launched, finalizing setup requires opening a terminal and connecting to the instance remotely via `ssh`. When connecting to the instance, the username `ec2-user` should be used, with the private key selected above and instance IP address, like follows:

```
$ ssh -i <path_to_private_key> ec2-user@<instance_public_ip>
```

A.1.2 Local Machine

The only required setup for running locally is to install Docker. Docker has installation helpers for all operating systems available on their website[7].

A.2 Getting Started with the ndmg Container

Prior to running the pipeline a directory to store the data must be created. This can be done as follows; here, a folder called `data` is created within the `HOME` directory.

```
$ mkdir ~/data
```

The ndmg container can then be downloaded with the following line:

```
$ docker pull bids/ndmg:v0.0.37
```

Note that this will download hundreds of MB of data to the machine.

A.3 Running Connectome Generation

Once the ndmg Docker container is downloaded the pipeline can be used. This step takes approximately 1-1.5 hours per scan (in this case, the NKI1 [30] dataset consists of 40 scans, which will take approximately 40-60 hours), so it is recommended that the following is executed within a `screen`[8] or similar type of session. The general structure of the command is as follows:

```
$ docker run -ti -v local_data:container_data bids/ndmg:version \
      input_data output_data analysis_level --bucket s3_bucket_name \
      --remote_path path_on_bucket
```

The command to run the pipeline is constructed from several choices. First, to access the derivatives produced within the pipeline, the data directory must be mounted to the container. As established in Section A.2, this is the /data directory. This directory can be mounted to /data within the container.

There are also several pipeline-specific parameters that need to be provided. These are: data input directory, data output directory, analysis level, and (optionally, but exercised here) the S3 bucket location and path to the data on said bucket. Table 2 enumerates the parameter selections for this demonstration. In summary, data is stored on an S3 bucket called mrneurodata, within the folder NKI24, and will be downloaded to the /data/raw folder locally. Connectome computation will be run (i.e. graphs and intermediate derivatives will be generated) and outputs stored in /data/connectome.

The pipeline can then be executed with the following command:

```
$ docker run -ti -v ~/data:/data bids/ndmg:v0.0.37 /data/raw \
      /data/connectome participant --bucket mrneurodata \
      --remote_path NKI24
```

Executing the above command will launch the container running the ndmg pipeline after retrieving data (organized in accordance to BIDS) from Amazon S3, and produce brain graphs in the location specified.

While the pipeline is executing it will produce verbose text output indicating which stage of the pipeline is being executed. The frequency of these updates ranges with the commands being executed, and spans approximately 30 seconds to 30 minutes depending on the point in the pipeline. If the pipeline were to fail, an error message would be displayed in the terminal indicating the problem. Once the job completes, a common desire is to visualize the derivatives produced; this can be done in the summary statistics step.

A.4 Running Summary Statistic Computation

The folder earlier assigned for derivatives for connectome generation now contains additional sub-folders, one of which is labeled graphs. Inside this folder are sub-folders for each of the parcellations used when generating the graphs. To generate Figure 4, the pipeline needs to be relaunched to compute summary statistics. The parameters are similar to the above, with the changes being analysis level, input data location, and the omission of the S3-related parameters. Table 3 summarizes these values.

The pipeline can again be run with the following command:

```
$ docker run -ti -v ~/data:/data bids/ndmg:v0.0.37 \
      /data/connectome/graphs /data/qc group
```

The folder assigned for this summary statistic or quality control storage will now contain a directory for each parcellation, and within that a file for each of several derivatives of the graphs, as well as an image and a JSON file which displays these metrics. The plot shown in Figure 4 can be found by opening ~data/qc/desikan/desikan_summary.png.

Appendix B Extension Instructions

As this is a living and breathing project undergoing development, changes are being made regularly. The reproduction instructions given in Appendix A will reproduce the exact results presented within this manuscript. There are several ways described below which enable staying up-to-date with the project and performing ones own analyses using this tool. All of the following instructions assume that the methods in Appendix A.1 have been executed.

B.1 Updating the ndmg Container

Re-pulling the container from Docker Hub using the tag `latest` enables using the most recent version of this tool.

```
$ docker pull bids/ndmg:latest
```

In order to use the newest version, the commands must be modified to replace the `:v0.0.37` version tag with `:latest`.

B.2 Using Your Data

The ndmg pipeline processes data according to the BIDS data specification. To use the tool with an alternate dataset, it first needs to be organized according to this specification. This can be validated using the BIDS Validator[9]. Once the data are organized, they can either be uploaded to an S3 bucket and processed with a command similar to that in Section A.3 (updating the bucket name and path to data on the bucket), or kept locally and omitting the `bucket` and `remote_path` values.

B.3 Changing the Parameters

All of the code for this project is open-source and resides in a Github repository[10]. To test the pipeline with different sets of parameters, it can be cloned and the source code can be modified directly. The repository can be cloned to the `HOME` directory with the following.

```
$ git clone https://github.com/neurodata/ndmg ~/ndmg
```

Once adjustments have been made and the new pipeline is ready to be tested, the package can be re-installed by executing the `setup.py` file contained within the repository.

```
$ cd ~/ndmg
$ python setup.py install
```

B.4 Changing the Functions

Much like changing parameters, once the repository is cloned it is possible to swap out algorithms or implementations for various parts of the pipeline. Examples of tools which could be replaced include registration or tractography. Again, once this is completed, the pipeline must be re-installed prior to execution.

Notes

[1] http://fcon_1000.projects.nitrc.org/indi/CoRR/html/nki_1.html
[2] http://brainx.io
[3] https://neurographics.net/2016/07/28/oden-2016/
[4] https://goo.gl/DDMcMG
[5] https://www.cygwin.com/
[6] For instructions on EC2 follow this guide: http://docs.aws.amazon.com/AWSEC2/latest/UserGuide/
[7] https://www.docker.com/products/overview
[8] https://www.gnu.org/software/screen/
[9] http://incf.github.io/bids-validator/
[10] https://github.com/neurodata/ndmg

Figure1

Click here to download Figure sic_framework.pdf

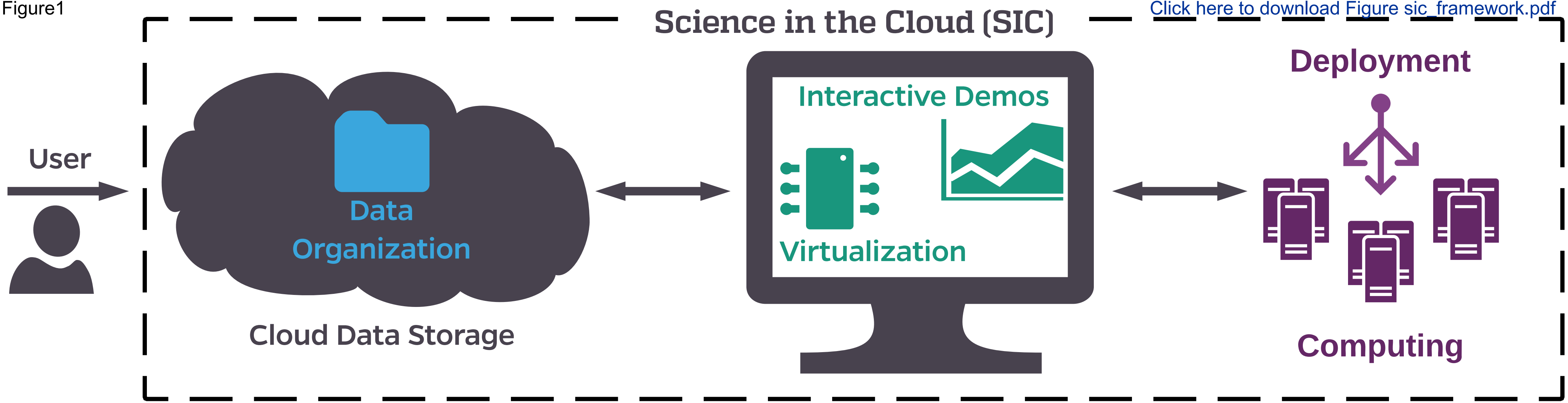

Figure2

Click here to download Figure ndmgworkflow.pdf

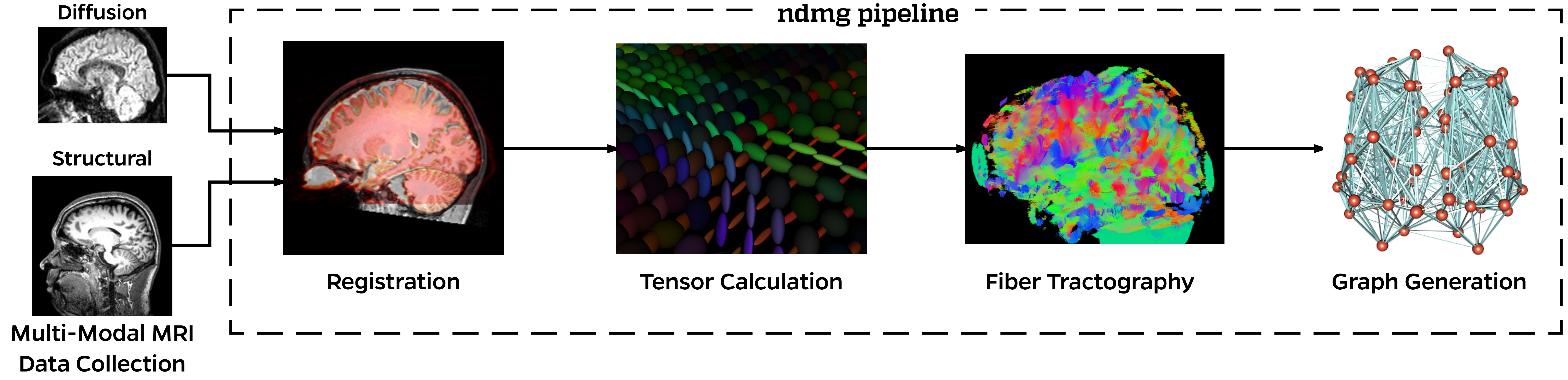

Figure3

Click here to download Figure Screenshot (10).png

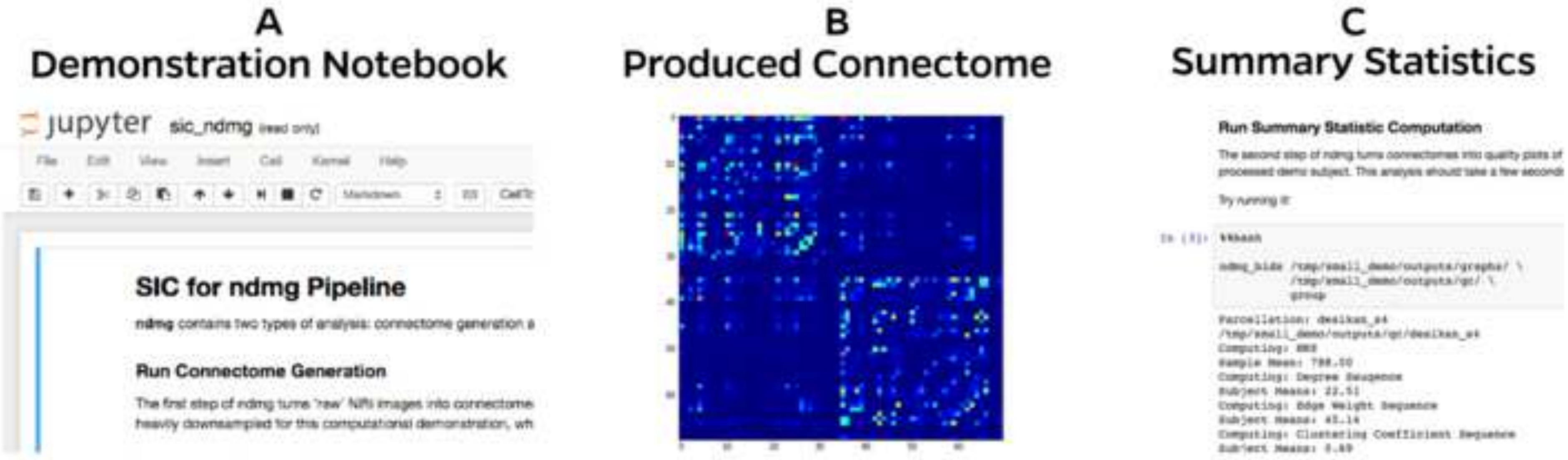

Figure4

Click here to download Figure NKI_desikan_summary.png

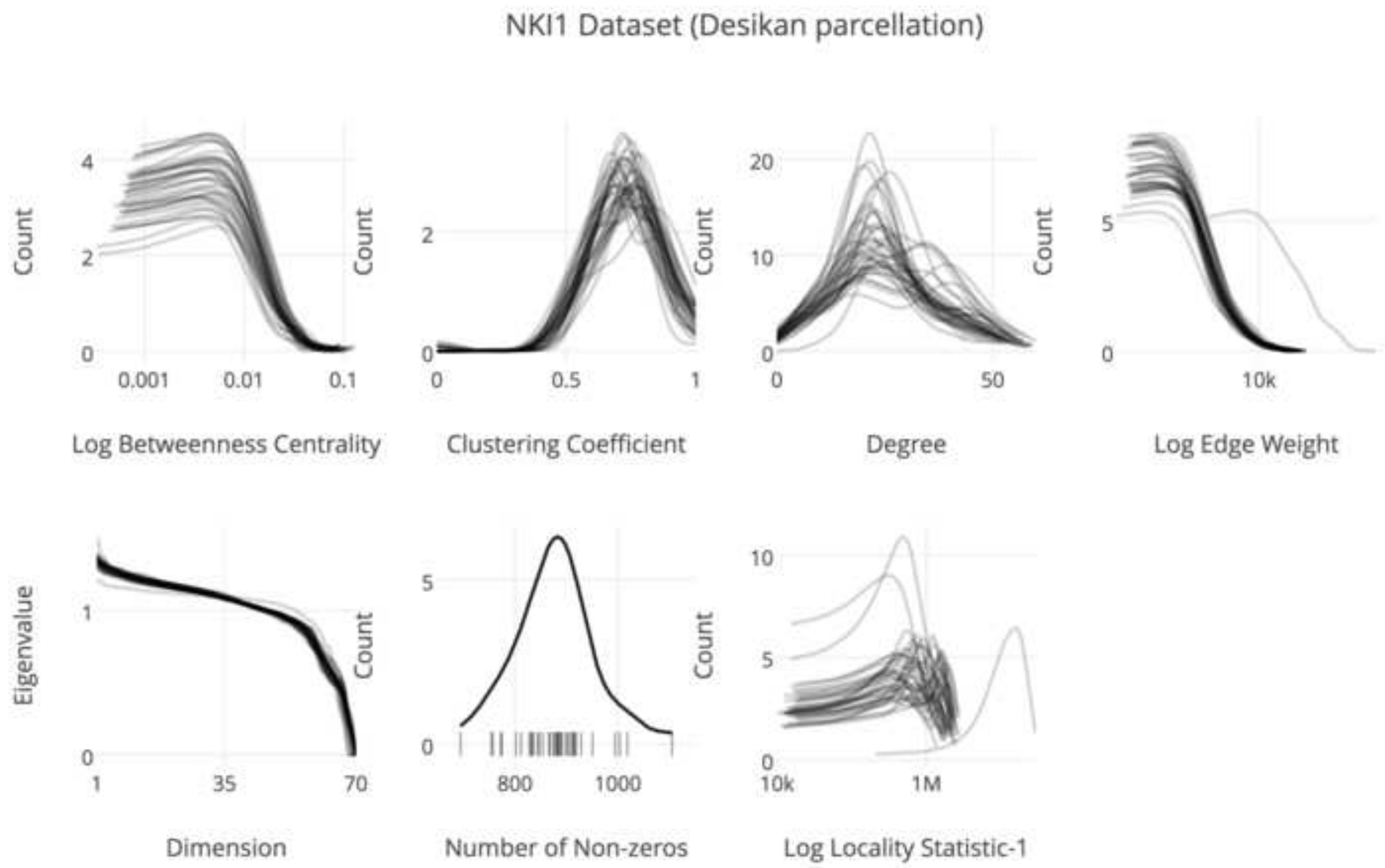

Figure5

Click here to download Figure KKI2009_desikan_summary.png

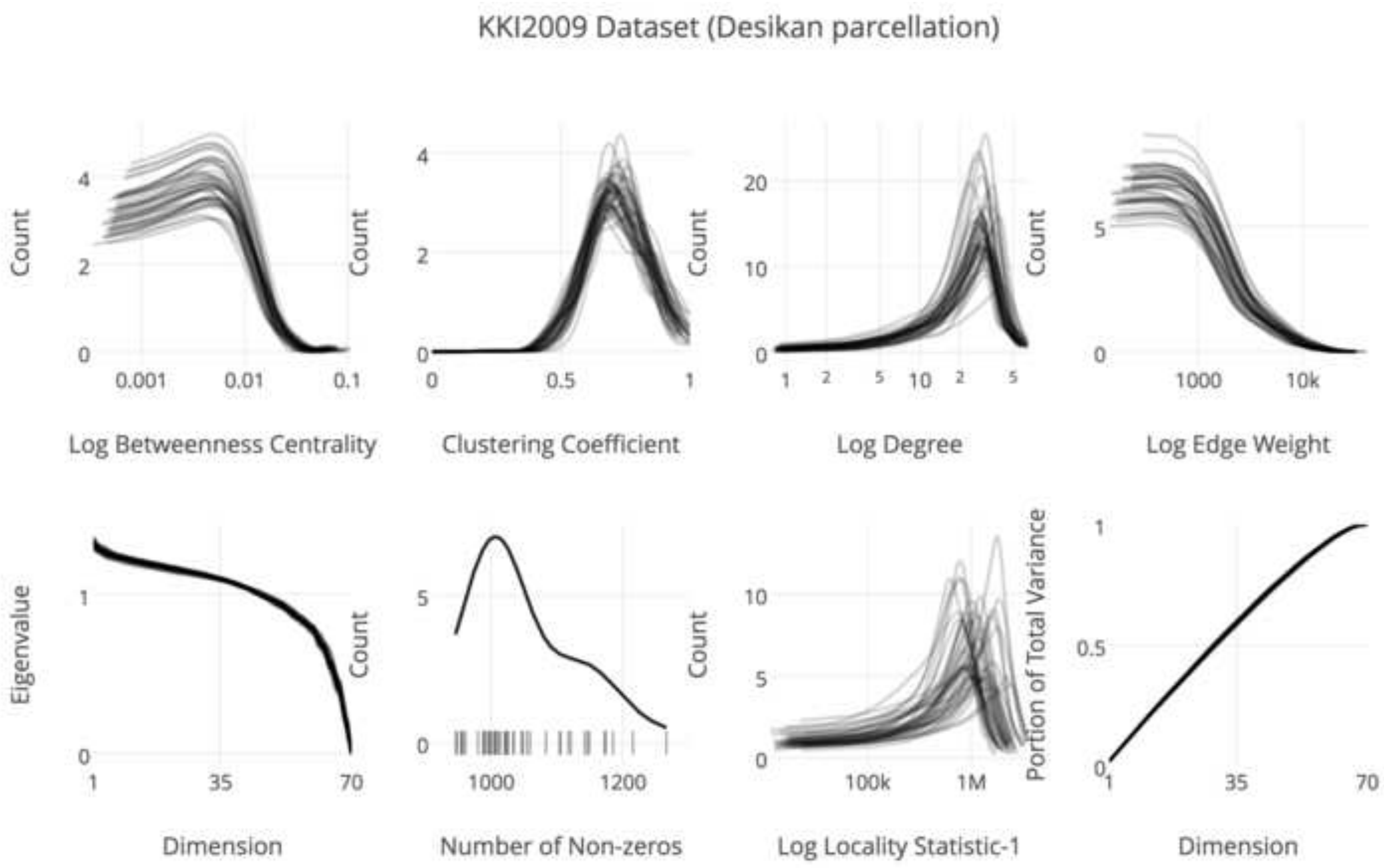

Supplement: GIGA-D-16-00143_Original_Submission.pdf [file gix013_GIGA-D-16-00143_Original_Submission.pdf]
