# Supplementary material for: Science in the cloud (SIC): A use case in MRI connectomics
Source: Gigascience. 2017 Mar 7;6(5):1–10. doi: 10.1093/gigascience/gix013 (PMC5467033; doi:10.1093/gigascience/gix013)
Supplement: Response_to_reviewer_comments_Original_Submission.pdf [file gix013_Response_to_reviewer_comments_Original_Submission.pdf]

Our responses to the provided feedback are enclosed within the cover letter attached in the following section alongside our manuscript.

In summary, we have made additions and modifications regarding all provided feedback, in particular: providing more background, discussing alternative strategies, using a parallel cloud deployment, and discussing how the tool can be extended to other tools and adopted by researchers.

Thank you very much for the terrific feedback, and we feel our manuscript is significantly better than the previous version. We are grateful to have an opportunity to re-submit our manuscript.
