# Supplementary material for: Science in the cloud (SIC): A use case in MRI connectomics
Source: Gigascience. 2017 Mar 7;6(5):1–10. doi: 10.1093/gigascience/gix013 (PMC5467033; doi:10.1093/gigascience/gix013)
Supplement: Response_to_reviewer_comments_Revision_1.pdf [file gix013_Response_to_reviewer_comments_Revision_1.pdf]

requested sections (funding, code resources, abbreviations) added; microsoft word version added.
