# Supplementary material for: Science in the cloud (SIC): A use case in MRI connectomics
Source: Gigascience. 2017 Mar 7;6(5):1–10. doi: 10.1093/gigascience/gix013 (PMC5467033; doi:10.1093/gigascience/gix013)
Supplement: Reviewer_1_Report_(Original_Submission).pdf [file gix013_Reviewer_1_Report_(Original_Submission).pdf]

Reviewer Report

Title: "Science In the Cloud (SIC): A use case in MRI Connectomics"

Version: Original Submission **Date:** 12/1/2016

Reviewer name: Peter Li

Reviewer Comments to Author:

Kiar et al. present a framework which they call, "Science In the Cloud (SIC)" for enabling reproducible analyses of data in neuroscience. The framework is comprehensive, providing a set of requirements describing how data should be shared and structured, and how the analysis of the data should be packaged for execution, extension and demonstration in an open and reproducible science manner. A use case is reported which shows a working example of the framework which involves a pipeline that processes M3RI data to generate a brain connectome graph.

I really like the thought that has gone into the framework especially using the Jupyter notebook for documenting how analyses are executed. The manuscript is well written and I was also able to reproduce the results of the use case using AWS with the instructions provided in Appendix A.

Its obvious a lot of effort has gone into the work described in the manuscript so my comments relate to the future of SIC. I think that a collection of neuroscience data analyses presented using the SIC framework would be a fantastic community resource. To this end, I am wondering how the authors will promote their work so it is adopted within the neuroscience science community. I couldn't find a web site showing the work in the manuscript which might be a useful thing to have. Perhaps http://scienceinthe.cloud could summarise the SIC framework?

Another barrier to adoption is that the SIC framework requires expertise in Cloud storage and computing, and virtualisation software in order to share neuroscience data analyses. I am wondering if a set of Software Carpentry-like lessons (http://software-carpentry.org/lessons) on these topics geared towards SIC might be worth thinking about developing in the future which could then be used as teaching materials in training workshops?

Minor correction

Fix typo on page 9 on the first line of the Discussion section: "The the…"

Peter Li

GigaScience

Methods

Are the methods appropriate to the aims of the study, are they well described, and are necessary controls included? Yes

Conclusions

Are the conclusions adequately supported by the data shown? Yes

Reporting Standards

Does the manuscript adhere to the journal's guidelines on minimum standards of reporting? Yes

Statistics

Are you able to assess all statistics in the manuscript, including the appropriateness of statistical tests used? There are no statistics in the manuscript.

Quality of Written English

Please indicate the quality of language in the manuscript: Acceptable

Declaration of Competing Interests

Please complete a declaration of competing interests, considering the following questions:

- Have you in the past five years received reimbursements, fees, funding, or salary from an organisation that may in any way gain or lose financially from the publication of this manuscript, either now or in the future?
- Do you hold any stocks or shares in an organisation that may in any way gain or lose financially from the publication of this manuscript, either now or in the future?
- Do you hold or are you currently applying for any patents relating to the content of the manuscript?
- Have you received reimbursements, fees, funding, or salary from an organization that holds or has applied for patents relating to the content of the manuscript?
- Do you have any other financial competing interests?
- Do you have any non-financial competing interests in relation to this paper?

If you can answer no to all of the above, write 'I declare that I have no competing interests' below. If your reply is yes to any, please give details below.

I am employed by GigaScience as a Data Organisation Manager.

I agree to the open peer review policy of the journal. I understand that my name will be included on my report to the authors and, if the manuscript is accepted for publication, my named report including any attachments I upload will be posted on the website along with the authors' responses. I agree for my report to be made available under an Open Access Creative Commons CC-BY license (http://creativecommons.org/licenses/by/4.0/). I understand that any comments which I do not wish to be included in my named report can be included as confidential comments to the editors, which will not be published.

I agree to the open peer review policy of the journal

To further support our reviewers, we have joined with Publons, where you can gain additional credit to further highlight your hard work (see: https://publons.com/journal/530/gigascience). On publication of this paper, your review will be automatically added to Publons, you can then choose whether or not to claim your Publons credit. I understand this statement.

Yes
