# Supplementary material for: Science in the cloud (SIC): A use case in MRI connectomics
Source: Gigascience. 2017 Mar 7;6(5):1–10. doi: 10.1093/gigascience/gix013 (PMC5467033; doi:10.1093/gigascience/gix013)
Supplement: Reviewer_2_Report_(Original_Submission).pdf [file gix013_Reviewer_2_Report_(Original_Submission).pdf]

Reviewer Report

Title: "Science In the Cloud (SIC): A use case in MRI Connectomics"

Version: Original Submission **Date:** 12/11/2016

Reviewer name: Sotirios Tsaftaris

Reviewer Comments to Author:

The authors present a neuroscience as a service solution. The manuscript is extremely well written, and presents a framework that could help several practitioners in the area of neuroinformatics and definitely aid reproducibility of results.

However, in its current form, it suffers from a few main issues (that some could be remedied):

a) Lack of a fair literature review. The way the authors present it, it appears they are the first to have attempted this.

For example, what is the relevance between what the authors present and:

* G. B. Frisoni, A. Redolfi, D. Manset, M.-E. Rousseau, A. Toga, and A. C. Evans, "Virtual imaging laboratories for marker discovery in neurodegenerative

diseases," Nature Reviews Neurology, vol. 7, no. 8, pp. 429-438, Jul. 2011.

* I. Dinov, K. Lozev, P. Petrosyan, Z. Liu, P. Eggert, J. Pierce, A. Zamanyan, S. Chakrapani, J. Van Horn, D. S. Parker, R. Magsipoc, K. Leung, B. Gutman,

R. Woods, and A. Toga, "Neuroimaging study designs, computational analyses and data provenance using the LONI pipeline," PLoS ONE, vol. 5, no. 9,

pp. e13 070+, Sep. 2010.

* neuGRID,

* outGRID

* the effort on NeuroDebian

* Neurodebian on AWS (EC2) https://www.nitrc.org/forum/forum.php?forum_id=3664

* M. Minervini, M. Damiano, V. Tucci, A. Bifone, A. Gozzi, S.A. Tsaftaris, "Mouse Neuroimaging Phenotyping in the Cloud," 3rd International Conference on Image Processing Theory, Tools and Applications, Special Session on Special Session on High Performance Computing in Computer Vision Applications (HPC-CVA) , Istanbul, Turkey, Oct 15-18, 2012.

* M. Minervini, C. Rusu, M. Damiano, V. Tucci, A. Bifone, A. Gozzi, S.A. Tsaftaris, "Large-Scale Analysis of Neuroimaging Data on Commercial Clouds with Content-Aware Resource Allocation Strategies," International Journal of High Performance Computing Applications, Jan 17, 2014.

I personally find relevance to the above methods at least in terms of motivation (albeit some may have used different methods). Obviously the last two were authored by my team a few years back, on the basis of a different Python based backbone that is now defunct (PiCloud). But the second one (last in the list), it went even beyond that: it considered optimization of resources (type of Amazon instance) with a machine learning method that predicted resource needs for non-linear registration in a pipeline of atlas based segmentation.

I am really fond of the approach of the authors as it adopts newer technologies (containers etc) that can perhaps make such systems future-proof. I should note that some of the technologies are used also by other systems on different applications. For example, there is US based initiative called CyVerse (iPlant) which the authors could explore as well.

b) Lack of discussion on how the current approach can be extended to use other tools such as freesurfer, ANTs etc

As I am sure you are aware, the same neuroimaging tools don't work for everyone. While I agree with the idea of having standardized pipelines, the ability to evolve said pipelines (as forks) can help the system evolve and (even) be maintained.

Can you please expand on this.

c) While the authors have cost estimates spread throughout the paper, I believe further discussion is necessary.

It would help the readers to understand for a typically sized study how much does it cost to upload data, store them for X days/months, download them, and for computation.

Based on our experience what was costly to store was the registration non-linear warps on the cloud and we had to keep special scripts to keep clean our data store.

Thus, perhaps it is advisable that the authors to include for the pipeline in Fig 2, who much time did each step take, how much did it cost, etc (maybe a table)?

d) Unfortunately, from at least how I understand the code, it appears that to do the same pipeline for the NKI1 dataset (40 scans) the process is linear (ie one scan after the others). This is enforced by the

comment of the authors in the discussion, related to Kubernetes, "would help enable SIC to scale well when working with big-data or running many parallel jobs. "

If this is true, the SIC framework loses on of the greatest aspects of cloud computing: that of scalability.

The authors should comment on this, particularly as this would make a proper fit for the GigaScience journal.

Minor comments:

First line of discussion, there is a double the.

Methods

Are the methods appropriate to the aims of the study, are they well described, and are necessary controls included? Yes

Conclusions

Are the conclusions adequately supported by the data shown? Yes

Reporting Standards

Does the manuscript adhere to the journal's guidelines on minimum standards of reporting? Yes

Statistics

Are you able to assess all statistics in the manuscript, including the appropriateness of statistical tests used? Yes, and I have assessed the statistics in my report.

Quality of Written English

Please indicate the quality of language in the manuscript: Acceptable

Declaration of Competing Interests

Please complete a declaration of competing interests, considering the following questions:

- Have you in the past five years received reimbursements, fees, funding, or salary from an organisation that may in any way gain or lose financially from the publication of this manuscript, either now or in the future?
- Do you hold any stocks or shares in an organisation that may in any way gain or lose financially from the publication of this manuscript, either now or in the future?
- Do you hold or are you currently applying for any patents relating to the content of the manuscript?

- Have you received reimbursements, fees, funding, or salary from an organization that holds or has applied for patents relating to the content of the manuscript?
- Do you have any other financial competing interests?
- Do you have any non-financial competing interests in relation to this paper?

If you can answer no to all of the above, write 'I declare that I have no competing interests' below. If your reply is yes to any, please give details below.

None

I agree to the open peer review policy of the journal. I understand that my name will be included on my report to the authors and, if the manuscript is accepted for publication, my named report including any attachments I upload will be posted on the website along with the authors' responses. I agree for my report to be made available under an Open Access Creative Commons CC-BY license (http://creativecommons.org/licenses/by/4.0/). I understand that any comments which I do not wish to be included in my named report can be included as confidential comments to the editors, which will not be published.

I agree to the open peer review policy of the journal

To further support our reviewers, we have joined with Publons, where you can gain additional credit to further highlight your hard work (see: https://publons.com/journal/530/gigascience). On publication of this paper, your review will be automatically added to Publons, you can then choose whether or not to claim your Publons credit. I understand this statement.

Yes
