# Supplementary material for: Science in the cloud (SIC): A use case in MRI connectomics
Source: Gigascience. 2017 Mar 7;6(5):1–10. doi: 10.1093/gigascience/gix013 (PMC5467033; doi:10.1093/gigascience/gix013)
Supplement: Reviewer_3_Report_(Original_Submission).pdf [file gix013_Reviewer_3_Report_(Original_Submission).pdf]

Reviewer Report

Title: "Science In the Cloud (SIC): A use case in MRI Connectomics"

Version: Original Submission **Date:** 12/13/2016

Reviewer name: Luis Bastiao Silva

Reviewer Comments to Author:

The article proposes a solution to solve a well-known problem in science, mainly in data science, which are the capability to reproduce experiences. As stated by the authors, the main problem is the lack of standardized for sharing mechanisms and applied practices often make reproducing scientific results very difficult. The authors rely on the several known and trend technologies and present a pipeline that could be used to tackle the problem and be reused by researchers to create reproducible experiments.

The manuscript is well written and follow a good presentation and structure, with a different and innovative methodology for the problem. In my vision, the main difficult to address the proposed pipeline, is the inherent complexity. For instance, while the authors propose the use of Docker containers to create easily setup scripts and data loading, in a real scenario there are two main criticisms: 1) the complexity of creating the Docker container by the research groups, for instance, considering the data scientists associated to the MRI problem may not have that knowledge; 2) to run the containers, it is still needed some technology background. Thus, the methodology and guidelines should be considered to approach the problem, and the strengths and weakness should be presented in discussion.

There are still some points that could be addressed in the methodology:

1. Data Storage: what kind of protocols should be considered? Only HTTP? If we considered to virtualize the machines, the users might want to have different access points and applied mount for instance, via NFS or CIFS. Moreover, could be another API used as for instance mount the Storage as a Volume?

2. Cloud environments: do you consider to use API middleware to solve the problem of different providers? There are libraries that allow to run machines from multiple clouds.

3. Docker: is proposed to run in AWS EC2 in the case study. But what are the differences between run in a local datacenter? Moreover, AWS has already a service dedicated to Docker containers. Could you consider to use this kind of tools in your approach? On the other hand, there are already tools like Totum that may facilitate the deployment of Docker containers. Could be a pre-installed machine help to deploy new containers?

4. Open standards for data: what are the standards and how they are used? It should be clarified in the manuscript.

5. Did you consider several levels of security? For instance, only allow the reviewers to access the container - online available?

6. What are the differences of this architecture comparing with only publishing a README with instructions? Easy for end-user, complex for developer/researcher.

7. Docker vs Vagrant? Could be a virtual machine do the same? What are the differences for the proposed pipeline? This kind of technical details should be addressed in the discussion, because in the end, the manuscript is placed as a technical research paper.

Methods

Are the methods appropriate to the aims of the study, are they well described, and are necessary controls included? Yes

Conclusions

Are the conclusions adequately supported by the data shown? No

Reporting Standards

Does the manuscript adhere to the journal's guidelines on minimum standards of reporting? Yes

Statistics

Are you able to assess all statistics in the manuscript, including the appropriateness of statistical tests used? No, and I do not feel adequately qualified to assess the statistics.

Quality of Written English

Please indicate the quality of language in the manuscript: Needs some language corrections before being published

Declaration of Competing Interests

Please complete a declaration of competing interests, considering the following questions:

- Have you in the past five years received reimbursements, fees, funding, or salary from an organisation that may in any way gain or lose financially from the publication of this manuscript, either now or in the future?
- Do you hold any stocks or shares in an organisation that may in any way gain or lose financially from the publication of this manuscript, either now or in the future?

- Do you hold or are you currently applying for any patents relating to the content of the manuscript?
- Have you received reimbursements, fees, funding, or salary from an organization that holds or has applied for patents relating to the content of the manuscript?
- Do you have any other financial competing interests?
- Do you have any non-financial competing interests in relation to this paper?

If you can answer no to all of the above, write 'I declare that I have no competing interests' below. If your reply is yes to any, please give details below.

I declare that I have no competing interests

I agree to the open peer review policy of the journal. I understand that my name will be included on my report to the authors and, if the manuscript is accepted for publication, my named report including any attachments I upload will be posted on the website along with the authors' responses. I agree for my report to be made available under an Open Access Creative Commons CC-BY license (http://creativecommons.org/licenses/by/4.0/). I understand that any comments which I do not wish to be included in my named report can be included as confidential comments to the editors, which will not be published.

I agree to the open peer review policy of the journal

To further support our reviewers, we have joined with Publons, where you can gain additional credit to further highlight your hard work (see: https://publons.com/journal/530/gigascience). On publication of this paper, your review will be automatically added to Publons, you can then choose whether or not to claim your Publons credit. I understand this statement.

Yes
